# Properties of Heat-Assisted pH Shifting and Compounded Chitosan from Insoluble Rice Peptide Precipitate and Its Application in the Curcumin-Loaded Pickering Emulsions

**DOI:** 10.3390/foods12244384

**Published:** 2023-12-06

**Authors:** Zhenyu Yang, Zhiying Li, Zitong Xu, Zhihao Kong, Xin Qiao, Liwen Zhang, Lei Dai, Yanfei Wang, Qingjie Sun, David Julian McClements, Xingfeng Xu

**Affiliations:** 1 College of Food Science and Engineering, Qingdao Agricultural University, 700 Changcheng Road, Chengyang District, Qingdao 266109, China; 19854280775@163.com (Z.Y.); lzyzyysd@163.com (Z.L.); 17568185502@163.com (Z.X.); kzh1026333@163.com (Z.K.); 19930659356@163.com (X.Q.); zlwzlwzlw2022@163.com (L.Z.); dailei508@163.com (L.D.); yanfeiwangqau@163.com (Y.W.); phdsun@163.com (Q.S.); 2 Qingdao Special Food Research Institute, Qingdao 266109, China; 3 Department of Food Science, University of Massachusetts, Amherst, MA 01003, USA; mcclements@foodsci.umass.edu

**Keywords:** chitosan, emulsion, insoluble peptide precipitates, interfacial properties

## Abstract

Curcumin exhibits antioxidant and antitumor properties, but its poor chemical stability limits its application. Insoluble peptide precipitates formed by proteolysis of rice glutelin are usually discarded, resulting in resource waste. The coupled treatment of heat-assisted pH shifting and compounded chitosan (CS) was used to fabricate rice peptide aggregate–chitosan complexes (RPA–CS). The structure, interfacial behavior, emulsion properties, and digestibility of curcumin-loaded RPA–CS Pickering emulsions were investigated. Increasing the CS concentration led to lower interfacial tension but larger particle size, and the three-phase contact angle of the RPA–CS complexes approached 90°. Quartz crystal microbalance with dissipation (QCM–D) indicated that RPA–CS complexes with 6 g·kg^−1^ of CS (RPA–CS_6_) had the highest K_1_ (0.592 × 10^6^ Hz^−1^) and K_4_ (0.487 × 10^6^ Hz^−1^), suggesting that the softest interfacial layers were formed. The solid–liquid balance of RPA–RPA–CS emulsions was lower than 0.5, declaring that they had more elastic behavior than that of RPA emulsions. RPA–RPA–CS_4_-and RPA–CS_6_ emulsions had better storage stability, lower FFA release (79.8% and 76.3%, respectively), and higher curcumin bioaccessibility (65.2% and 68.2%, respectively) than RPA emulsions. This study showed that a low-value insoluble rice peptide precipitate could be used as a valuable emulsifier in foods, which may increase the economics and sustainability of the food supply.

## 1. Introduction

Glutelin is commonly used as a plant-based functional protein due to its low allergenicity and high nutritional value, but the high hydrophobicity limits its application. Previously, limited enzymatic hydrolysis enhanced the water solubility and emulsion stability of rice glutelin [1]. However, a number of non-polar peptides were generated during the hydrolysis of protein [2], and then insoluble peptide precipitates with poor functional properties tended to form. These peptide precipitates are usually used as animal feed or discarded, leading to a serious waste of resources. Therefore, improving the functional attributes of insoluble peptide precipitates and increasing their utilization are very important.

The heat-assisted pH-shift method is a useful way of enhancing the functional properties of proteins. During the pH-shifting process, the protein is initially subjected to extreme alkaline pH to obtain a molten state, and then the pH value is returned to the neutral environment using an acidic solution. Furthermore, temperature treatment during the pH-shifting process was reported to promote the functional properties of the protein. The solubility and emulsifying properties of pea protein were significantly improved by heat-assisted pH-shift treatment, and pea protein nanoparticles were formed [3]. Additionally, a depolymerized tertiary structure, exposed hydrophobic groups, and enhanced emulsion were found for egg white protein modified by mild heating-assisted pH 12 shifting [4]. Therefore, we speculated that insoluble rice peptide precipitates treated with heat-assisted pH 12 shifting would be a promising nanoparticle emulsifier ingredient for stabilizing emulsions.

Curcumin is a natural phenolic substance originating from the rhizome of turmeric, which exhibits antioxidant, anti-inflammatory, and antitumor properties. But its strong hydrophobicity and poor chemical stability make it challenging to utilize as a nutraceutical in the food industry [3]. These challenges can often be overcome by encapsulating curcumin within colloidal particles, such as Pickering emulsions. Protein-based Pickering emulsions are one of the widely used platforms for creating colloidal delivery systems for application in the food industries. However, protein-based Pickering emulsions are not only sensitive to environmental factors [5] but also have poor performance in gastrointestinal digestion, such as low bioavailability of bioactive compounds [6]. The exploitation of protein and polysaccharide complexes displays opportunities for the fabricating of a novel Pickering emulsion stabilizer. Chitosan (CS) is a cationic polysaccharide with good biocompatibility and biodegradability [7]. Protein–CS complexes showed better ability in stabilizing emulsions because of the interaction between cationic groups on CS and anionic groups on proteins. Potato protein–CS complex emulsions had better stability and lower digestibility than those stabilized by potato proteins alone [8]. The encapsulation of β-carotene in phosphorylated perilla protein isolate–CS emulsions was higher than that of without CS after environmental stress treatment [9]. However, these studies mainly focused on the interaction of protein and CS and the stability of emulsions prepared by soluble protein molecule–CS complexes. The effect of the coupled treatment of heat-assisted pH shifting and compounded CS on the properties of emulsions stabilized by insoluble rice peptide precipitate is currently lacking.

The present study intended to explore the effect of heat-assisted pH shifting and compounded CS on the physicochemical properties, microrheology, stability, and in vitro digestive properties of insoluble rice peptide precipitate emulsions. This research would provide relevant information for the application of insoluble rice peptide precipitates as a novel emulsifier and delivery system in the food industry.

## 2. Materials and Methods

### 2.1. Materials

Rice glutelin (900 g·kg^−1^) was supplied by Jiangxi Gabio Technology Co., Ltd. (Yichun China). Nile red, FITC, chitosan (CS, deacetylation degree ≥ 95%, viscosity 100–200 m·Pas), mucin, curcumin, trypsin (T8150), porcine bile salt (G8310), pepsin (P8160), and porcine lipase (L8070, Type II) were provided by Beijing Solarbio Technology Co. Ltd. (Beijing, China). All other reagents were of analytical grade.

### 2.2. Preparation of Insoluble Peptide Precipitate and Rice Peptide Aggregate (RPA)

60 mg·mL^−1^ of rice glutelin solution was adjusted to pH 8.0 and kept at 50 °C for 30 min. Then, trypsin was used for hydrolysis, and inactivation of the enzyme was carried out at 90 °C for 10 min. Then, the mixed suspension was centrifuged (3000 r·min^−1^, 15 min) [1]. The precipitate was collected and freeze-dried, which was taken to be the insoluble peptide precipitate.

Our pre-experiment result showed that the insoluble peptide precipitate (30 g·kg^−1^) had better solubility when treated at pH 12 (90 °C, 2 h) and readjusted to pH 7. Therefore, the treated insoluble peptide precipitate dispersion was centrifugated at 10,000 r·min^−1^ for 30 min, and the supernatants were collected, dialyzed, and freeze-dried for further experiments. The obtained sample was referred to as rice peptide aggregate (RPA).

### 2.3. Preparation of Rice Peptide Aggregate–Chitosan (RPA–CS) Complexes

The RPA–CS complex was prepared following the method of Hu et al. [8] with a slight modification. The RPA (20 g·kg^−1^) and chitosan (CS, 12 g·kg^−1^) were dissolved in distilled water and (1 L·100 L^−1^) acetic acid solution, respectively, to obtain stock solutions. The RPA–CS complexes with a range of CS concentrations (0, 2, 4, and 6 g·kg^−1^) were prepared by mixing the RPA and CS stock solutions in equal volumes. The final concentration of RPA in the mixed system was 10 g·kg^−1^ (pH 3). The samples with different concentrations of CS were named RPA, RPA–CS_2_, RPA–CS_4_, and RPA–CS_6_, respectively.

### 2.4. ζ-Potential, Turbidity, and Z-Average Diameter of RPA–CS Complexes

The freshly prepared complex was adjusted to different pH values (pH 2–11) using 0.1 mol·L^−1^ HCl and NaOH [10]. The ζ–potential and Z–average diameter were measured using Zetasizer Nano (Malvern Instrument Co., Ltd., Shanghai, China). The turbidity was analyzed at 600 nm by an ultraviolet spectrophotometer (Shanghai Yuanxi Instrument Co., Ltd, Beijing, China). All measurements were performed in triplicate.

### 2.5. Fourier Transform Infrared Spectroscopy (FTIR) 

The absorbance spectra of samples were collected from 4000 to 400 cm^−1^ (Thermo, Waltham, MA, USA) [11]. RPA–CS complex powder was blended with KBr powder at a ratio of 1:100, and then the mixtures were compressed into pellets. The resolution parameter and scan number were set to 4 cm^−1^ and 32 times, respectively.

### 2.6. Scanning Electron Microscopy (SEM)

The morphology was obtained using an SU8010 SEM (JEOL, Beijing, China). Frozen samples were affixed to a tape and sprayed with a thin gold layer before observation, and then scanned and photographed at 5 kV.

### 2.7. Interfacial Properties

#### 2.7.1. Wettability Measurement

The freeze–dried samples were pressed into tablets and immersed in corn oil. Afterward, ultrapure water (5 μL) was deposited onto the tablet surface [12]. Images were collected after 2 min equilibrium using a CCD camera system, and then the three–phase contact angle was evaluated on the basis of the Young–Laplace equation.

#### 2.7.2. Dynamic Interfacial Tension

The dynamic interfacial adsorption process was characterized using DSA100 (Krüss GmbH, Hamburg, Germany) [13]. A total of 45 μL of the sample solution was injected into the corn oil phase and the whole process lasted for 10,800 s. Interfacial tension data were obtained through the drop shape analysis software (DSA1 v1.9).

#### 2.7.3. Quartz Crystal Microbalance with Dissipation (QCM–D) Experiments

The interfacial adsorption behavior of RPA–CS complexes was carried out using QCM–D (Biolin Scientific, Gothenburg, Sweden) [14]. A cleaned sensor was submerged in 1-hexadecanethiol to form a self-assembled hydrophobic layer. The double-distilled water (pH 3) was injected at a flow rate of 0.1 mL·min^−1^, and then the test solution (10 g·kg^−1^) was pumped into the chamber to obtain the frequency and dissipation information. Lastly, the unbounded samples were washed off by double–distilled water (pH 3) after 90 min. The Sauerbrey model was used to calculate the interfacial thickness [15].

### 2.8. Fabrication of Curcumin–Loaded Pickering Emulsions

Curcumin–loaded Pickering emulsions were prepared according to previous research [3]. 1 g·kg^−1^ curcumin was dispersed in corn oil and incubated at 85 °C for 2 h. RPA–CS complex dispersion (900 g·kg^−1^) was mixed with the oil phase (100 g·kg^−1^). The mixture was sheared by a high-speed mixer at 18,000 r·min^−1^ for 2 min firstly and then sonicated at 450 W for 5 min to form the fine emulsion. Finally, sodium azide (0.2 g·kg^−1^) was used to prevent microbial contamination.

### 2.9. Storage Stability of Curcumin–Loaded Emulsions

To evaluate the storage stability, curcumin–loaded emulsions were stored at 25 °C for 7 days protected from light. The surface-weighted mean diameter (*d_3,2_*) was measured using Mastersizer 3000 (Malvern Instrument Co., Ltd., China). The ζ–potential was obtained by Zetasizer Nano (Malvern Instrument Co., Ltd., China). All samples were dispersed in ultrapure water with a pH of 3.

The polydispersity index (PDI) was calculated as follows [16]:

(1) $$\mathrm{PDI}=\frac{D_{0.9}-D_{0.1}}{D_{0.5}}$$

Here, D_0.1_, D_0.5_, and D_0.9_ were the diameters at 10%, 50%, and 90% cumulative volume, respectively. Put another way, (D_0.9_–D_0.1_) is the range of the data and D_0.5_ is the median diameter.

The protein and oil phases were labeled by Nile red and FITC (1 mg·mL^−1^), respectively. The emulsion (5 μL) was placed on a microscope slide, which was covered with a coverslip. A confocal laser scanning microscopy (CLSM) image was observed using a Leica TCS SP5. The Nile red and FITC were excited at 488 nm and 543 nm, respectively [17].

### 2.10. Microrheological Properties of Curcumin-Loaded Emulsions

The microrheological properties were determined using a Rheolaser Master (Formulaction, Toulouse, France) [18]. Four milliliters of fresh emulsion were put in a cylindrical glass tube and then the Brownian motion of the droplets was monitored immediately. The data were expressed as mean square displacement (MSD), elasticity index (EI), and macroscopic viscosity index (MVI) using software (RheoSoft Master 1.4.0.0).

### 2.11. In Vitro Digestion

The emulsions’ digestibility was measured as described previously [19]. The droplet characteristics were determined after each stage of in vitro digestion. The release rate of free fatty acid was obtained as follows [20]:

(2) $$\mathrm{FFA}\;\mathrm{release}\;\mathrm{rate}\left(\%\right)=\frac{V_{\mathrm{NaOH}}\times M_{\mathrm{NaOH}}\times M_{\mathrm{oil}}}{{2\times W}_{\mathrm{oil}}}\times 100\%$$

Here, V_NaOH_ and M_NaOH_ were the volume and molarity (0.15 mol·L^−1^) of NaOH solution consumed in neutralizing the FFAs produced, respectively; M_oil_ was the molecular weight of the corn oil (800 g·mol^−1^); W_oil_ was the total weight of the initial corn oil presented in the reaction vessel (0.15 g).

The bioaccessibility (%) and transformation (%) of curcumin were obtained according to a previous study using the equations below [19]:

(3) $$\mathrm{Bioaccessibility}\left(\%\right)=\frac{C_{\mathrm{micelle}}}{C_{\mathrm{initial}\;\mathrm{emulsion}}}\times 100\%$$

(4) $$\mathrm{Transformation}\left(\%\right)=\frac{C_{\mathrm{digesta}}}{C_{\mathrm{initial}\;\mathrm{emulsion}}}\times 100\%$$

where C_micelle_, C_digesta_, and C_initial emulsion_ were the contents of curcumin determined in the micelle fraction, small intestine phase, and the original emulsion sample, respectively.

### 2.12. Statistical Analysis

All measurements were conducted in triplicate. Statistical analyses were calculated as means and standard deviations. A Duncan test was used in the analysis of variance to determine significant differences (ANOVA, *p* < 0.05).

## 3. Results and Discussion

### 3.1. Effect of pH on the RPA–CS Complexes

#### 3.1.1. Turbidity

**Figure 1:**
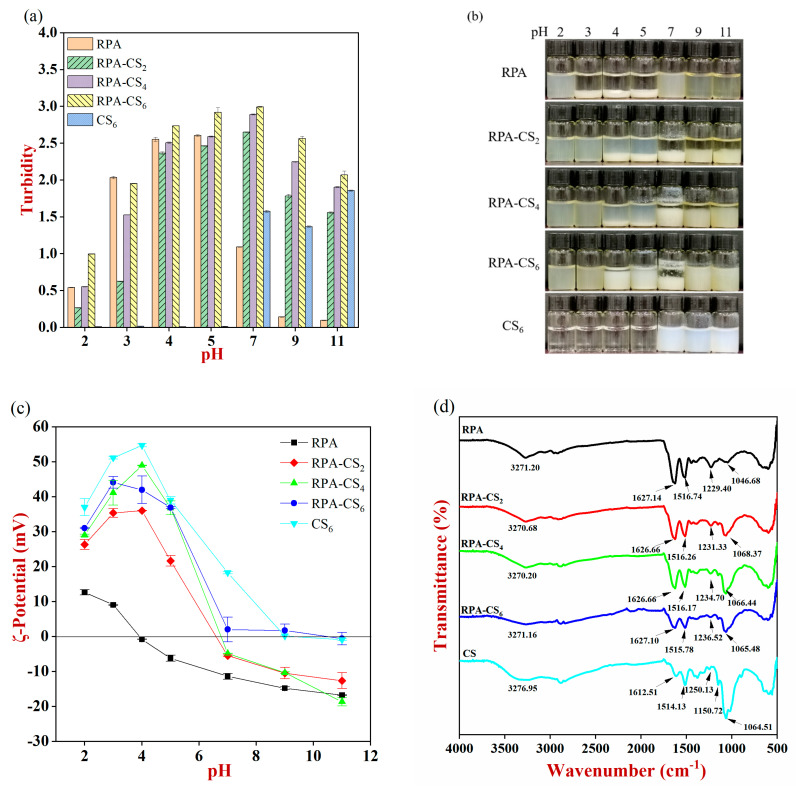

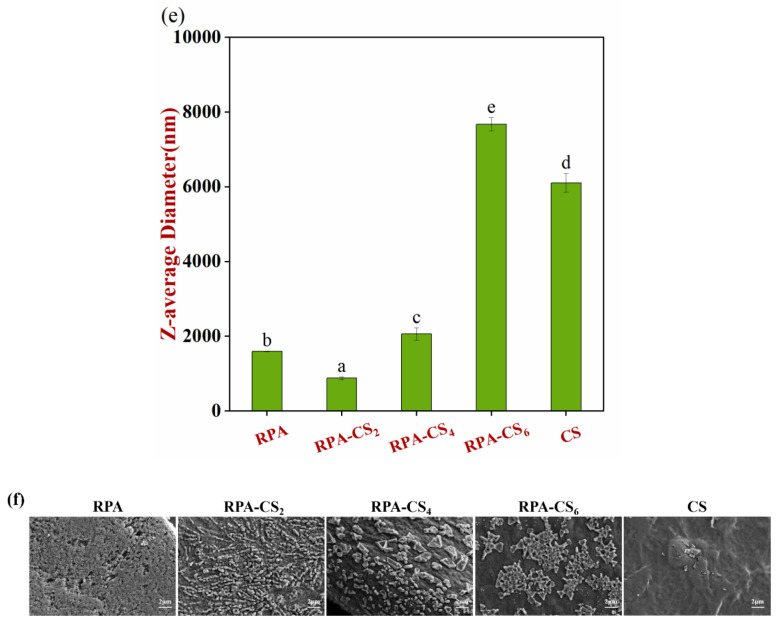
(**a**) The turbidity of samples, (**b**) appearance diagram after 24 h of storage, (**c**) ζ–potential at different pH values, (**d**) FTIR spectra, (**e**) z–average diameter (Dz), and (**f**) microstructures of the RPA, CS, and RPA–CS complexes. Different letters represent significant differences between groups (*p* < 0.05).

Precipitates were clearly observed at the bottom of the bottle over the pH range for insoluble peptide precipitate samples (Figure S1), which might be due to the formation of disulfide bonds and/or hydrophobic interactions between the protein subunits [1]. A high turbidity could be found for RPA from pH 3 to 5 (Figure 1a), which was consistent with the visual observations (Figure 1b). However, a relatively stable cloudy colloidal dispersion was observed for RPA in other pH ranges, which could be due to the strong electrostatic repulsion inhibiting the aggregation of the peptide molecules. A similar result was also reported by Hu et al. [8]. These results suggested that the heat–assisted pH–shift method could improve the dispersibility of the insoluble peptide precipitates.

The turbidity of the RPA–CS complexes increased first and then decreased across the pH range. A uniform suspension could be observed for RPA–CS complexes at pH 2 and 3. This could be attributed to the following reasons: Firstly, the electrostatic repulsion between RPA and CS might prevent the intermolecular aggregation. Secondly, some soluble complexes might be formed due to the interaction of anionic regions of RPA and cationic groups on the CS. When the pH was higher than 4, phase separation could be found for RPA–CS complexes (Figure 1b). This behavior suggested that there was a strong electrostatic attraction between the peptides and polysaccharide molecules. In addition, the reduced solubility of CS could also cause an increase in turbidity [21]. The turbidity of the RPA–CS complexes decreased appreciably when the pH was larger than 7. This was probably because the positive charge on the CS molecules was reduced due to the deprotonation of the amino groups, thereby decreasing their tendency to be attracted to the anionic peptides [22]. The turbidity of whey protein/CS complexes also increased at first and then declined with the rise in pH [23].

#### 3.1.2. ζ–Potential Analysis

The ζ-potential of RPA changed from +12.7 mV to −16.8 mV when the pH was raised from 2 to 11, with a point of zero change near pH 4 (Figure 1c). This behavior was consistent with the visual observations, which showed that phase separation occurred for RPA from pH 3 to 5, presumably due to the low electrostatic repulsion between them. The ζ–potential of CS was positive during pH 2 to 7, with a maximum value at pH 4. The relatively high positive charge could prevent the aggregation of CS molecules by generating a strong electrostatic repulsion [24], leading to a clear solution with low turbidity. All the RPA–CS complexes exhibited fairly similar trends across the entire pH range. The ζ–potential was relatively high for RPA–CS complexes at pH 3 and 4, demonstrating the intensity of interaction forces between RPA and CS [24].

In summary, a relatively stable cloudy colloidal dispersion was found for RPA–CS complexes at pH 3, which is relatively close to the pH of acidic beverages. Therefore, the RPA–CS complexes at pH 3 were used for the subsequent studies.

### 3.2. Fourier Infrared Spectroscopy

The main absorption bands of CS were observed at 3276, 1612, and 1065 cm^−1^ (Figure 1d), which were assigned to the stretching vibrations of N-H, C=O, and C-O-C, respectively [21]. New peaks appeared for RPA–CS_2_ (1068 cm^−1^), RPA–CS_4_ (1066 cm^−1^), and RPA–CS_6_ (1065 cm^−1^), which was due to the introduction of CS. Peaks in 3400–3200 cm^−1^ (amide A) were related to hydrogen bonding, O-H, and N-H stretching vibrations. The peaks of RPA–CS in the amide A band shifted from 3270.68 to 3271.16 cm^−1^ with the rise in CS concentration, indicating the presence of hydrogen bonding interactions between RPA and CS. The amide I band (1700–1600 cm^−1^) corresponded to the stretching vibrations of C=O, which corresponded to the secondary structure of protein [25]. The spectrum of RPA–CS complexes exhibited changed position and intensity as compared to those of RPA, demonstrating that the secondary structure of the RPA was altered by CS interaction. The addition of CS also led to changes in the conformation of whey protein [23,26] The peaks in the amide II band (1550–1500 cm^−1^) were assigned to N-H bonding, and the adsorption peaks in the region of the amide III band (1450–1200 cm^−1^) corresponded to stretching vibrations of C-N and N-H stretching [27]. The amide II band of the RPA–CS complexes was slightly moved from 1516.74 to 1515.78 cm^−1^, while the amide III band was shifted from 1229.40 to 1236.52 cm^−1^ after the addition of CS. This behavior was attributed to the changes in the N-H and C-N groups’ stretching vibrations. Zhao et al. also showed that the changes in peak intensity in the amide III band were due to the electrostatic interaction between anionic regions of protein and cationic groups on the CS, leading to the formation of soluble complexes [9].

### 3.3. Characterization of RPA–CS Complexes

The Z-average diameter of RPA decreased at first and then increased with the addition of CS (Figure 1e). For instance, the Z-average diameter of RPA, RPA–0.2%CS, RPA–0.4%CS, and RPA–0.6%CS were 1598, 880, 2026, and 7676 nm, respectively. The particle diameter of the RPA–CS_2_ complexes was less than that of RPA or CS alone, suggesting that there was a change in the conformation and/or aggregation state of the biopolymers after complexation. Ding et al. also suggested that the shrinkage of polysaccharide chains occurred when the interaction of WPI and CS occurred at a certain biopolymer proportion [23]. Additionally, the relatively high electrical charge of RPA–CS complexes led to a strong electrostatic repulsion, which inhibited their aggregation, leading to a smaller Z–average diameter. At higher CS concentrations, multiple CS molecules might have been attached to each peptide molecule, leading to an increase in particle diameter [23]. This result was consistent with the SEM images shown in Figure 1f. These images show that more aggregation of the RPA–CS complexes occurred as the CS concentration increased. In particular, the RPA–CS_6_ complexes were present as large aggregates with irregular morphologies. The Z–average diameter of phosphorylated perilla protein–CS particles also increased with increasing CS concentration [9].

### 3.4. Interfacial Properties

#### 3.4.1. Wettability Analysis of RPA–CS Complexes

Wettability analysis was used to evaluate the hydrophilic–hydrophobic properties of particles, as this impacts their tendency to anchor to oil–water interfaces and stabilize Pickering emulsions. The three–phase contact angle of the particles at an oil–water interface was used to characterize their wettability. The three-phase contact angle of the particles at an oil–water interface was used to characterize their wettability. The RPA had a contact angle of 68°, indicating that it was hydrophilic. As the CS concentration increased, the three-phase contact angle of the RPA–CS complexes approached 90°. This result was coincident with that of Li et al., showing that the three-phase contact angle of the complex increased with the addition of CS. This might be achieved by the hydrophobically modified CS [28].

#### 3.4.2. Interfacial Tension Analysis of RPA–CS Complexes

**Figure 2:**
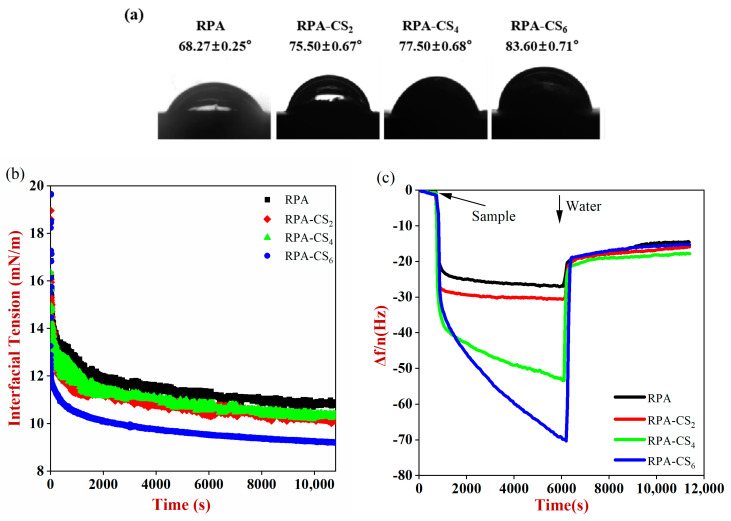

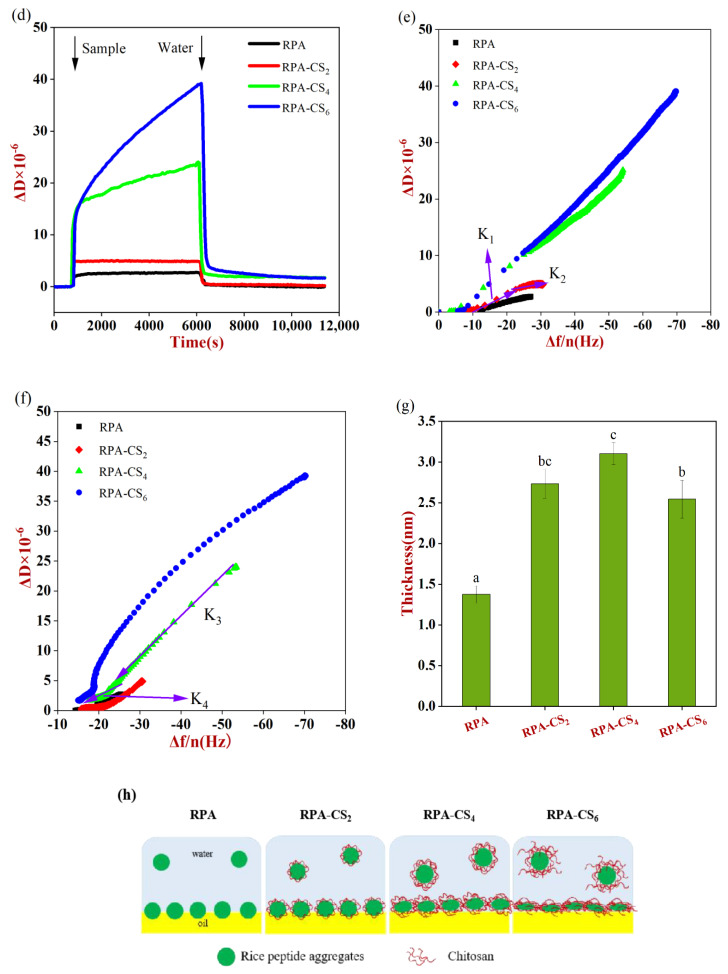
(**a**) The three–phase contact angle and (**b**) interfacial tension of samples. The (**c**) Δf/n and (**d**) ΔD versus time due to samples’ adsorption and desorption on the hydrophobic gold surface. ΔD–Δf/n plots for the (**e**) adsorption and (**f**) desorption processes (K_1_, K_2_, K_3_, and K_4_ denote the slopes of the four different phases, respectively). (**g**) The thickness of the samples adsorbed on the hydrophobic surface after rinsing. (**h**) Schematic diagram of the conformational changes of RPA and RPA–CS complexes at oil–water interface. Different letters represent significant differences between groups (*p* < 0.05).

The interfacial tension of RPA–CS complexes was measured to investigate their affinity for oil–water interfaces. The interfacial tension of all the samples declined with the extension of time (Figure 2b), suggesting that the samples were adsorbed to the oil–water interface. Compared with RPA, the interfacial tension of the RPA–CS complexes was relatively low and positively correlated with CS concentration. This suggested that the presence of CS enhanced the surface activity of the RPA–CS complexes. The result was consistent with the finding of Zhao et al. [9]. The presence of the hydrophobic CS might increase the hydrophobicity of the complexes, thereby promoting their tendency to adsorb to interfaces. Moreover, the -NH_3_^+^ groups in the CS may have been electrostatically attracted to the -COO^−^ groups in free fatty acids at the oil droplet surfaces, leading to the decline in interfacial tension [29]. This result was therefore consistent with the wettability results discussed earlier.

#### 3.4.3. Adsorption of RPA–CS Complexes on Hydrophobic Surfaces

QCM–D was used to investigate the adsorption of the proteins and their complexes to an interface. Changes in frequency (Δf/n) and dissipation (ΔD) provide information about the thickness and rheology of adsorbed substances, respectively. The decrease in Δf/n and increase in ΔD over time (Figure 2c,d) suggested the occurrence of adsorption [30]. The dissipation values of RPA–CS_2_ and RPA–CS_4_ increased sharply, indicating that they formed a viscoelastic layer at the oil–water interface. The addition of carboxymethyl chitosan to whey protein also promoted the formation of the softest viscoelastic layer [31]. When water (pH 3.0) flowed across the surface of the sensor, Δf/n increased and ΔD decreased until relatively constant values were reached. The phenomenon declared that some of the proteins and complexes were strongly attached to the surfaces, but others were only loosely attached and could be removed by rinsing [32].

ΔD–Δf curves are used to provide information about the conformational changes within adsorbed layers. If there is an inflexion point in the ΔD–Δf curve during the whole process, then the interfacial layer undergoes appreciable conformational changes. Conversely, if ΔD–Δf does not exhibit an inflexion point, then the adsorbed layer does not undergo an appreciable conformational change. In general, a smaller slope indicates a more rigid interfacial layer, whereas a higher slope indicates a more viscous interfacial layer. The ΔD–Δf/n plots were constant for RPA, RPA–CS_4_, and RPA–CS_6_ during adsorption (Figure 2e), indicating that there was no conformational change during adsorption. During the rinsing process, the ΔD–Δf curve of all samples had an inflexion point, indicating that a conformational alteration had occurred. The highest K_1_ and K_4_ values for RPA–CS_6_ manifested that the softest interfacial layers were formed (Table 1). The lowest K_4_ slope for RPA declared its more rigid layers. After rinsing, the viscoelasticity of the interfacial layer enhanced with increasing CS concentration. This was coincident with the results of Zhao et al., who studied phosphorylated perilla protein–CS systems [9].

The thicknesses of RPA, RPA–CS_2_, RPA–CS_4_, and RPA–CS_6_ layers absorbed onto the hydrophobic gold surface after rinsing were 1.38, 2.74, 3.11, and 2.55 nm, respectively (Figure 2g). Interestingly, RPA–CS_6_ exhibited a higher Z-average diameter but a lower adsorption thickness than RPA–CS_4_. This might be because the layer formed by RPA–CS_6_ was less able to protect the particles from aggregation. Additionally, the soft RPA–CS_6_ particles more often tended to deform at the oil–water interface, leading to a partial decrease in interfacial layer thickness (Figure 2h). The results in Table 1 also show that RPA–CS_6_ formed softer and thinner layers than the other samples. β-lactoglobulin–propylene glycol alginate complex nanoparticles also deformed and spread out when anchored onto the interface surface [33].

### 3.5. Storage Stability Analysis of Emulsions

**Figure 3:**
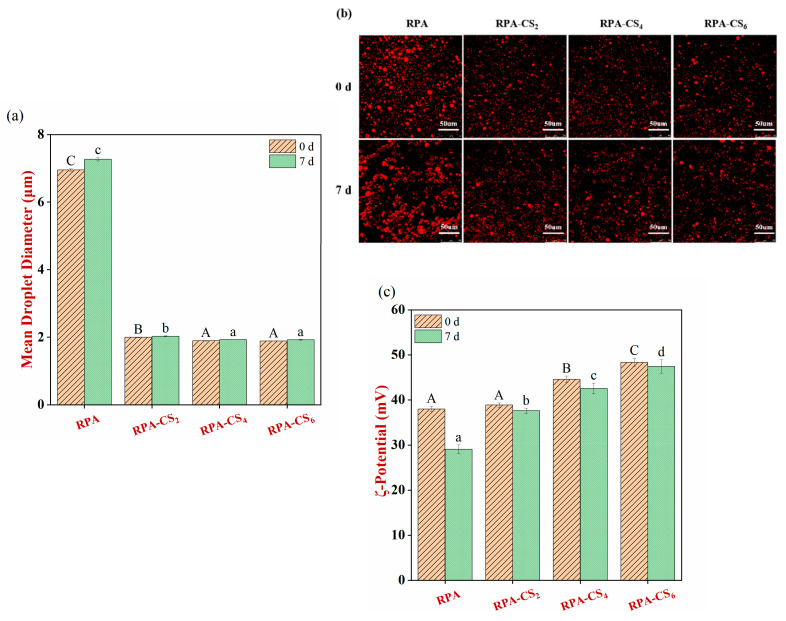
(**a**) The mean droplet diameter (*d_3,2_*), (**b**) confocal microscopy images, and (**c**) ζ-potential of the Pickering emulsions stabilized by RPA and RPA–CS complexes after 0 days and 7 days of storage. Different capital letters (A, B, C) and different low-case letters (a, b, c, d) indicate significant difference between different samples at 0 day and 7 days, respectively.

The fresh RPA–stabilized emulsion had a relatively large *d_3,2_* of around 7.0 μm (Figure 3a). This phenomenon might be due to the poor solubility of RPA at pH 3.0. As a result, the RPA did not rapidly adsorb to the droplet surfaces during homogenization and was not effective at protecting the oil droplets from aggregation after homogenization. The RPA–CS presented a reduced *d_3,2_* (with a more uniform size, Figure S2) and an increased positive charge with the addition of CS. This might be because the presence of CS enhanced the steric and electrostatic repulsion between the oil droplets, thereby inhibiting their aggregation. The *d_3,2_* of emulsions stabilized by potato protein/CS complexes also decreased after the addition of CS [8].

The *d_3,2_* of the RPA–stabilized emulsion increased with creaming and absolute ζ–potential declined after 7 days of storage, indicating that there was a slight increase in droplet aggregation during storage due to some alterations in interfacial composition [34]. This phenomenon was confirmed by CLSM analysis (Figure 3b). In contrast, the *d_3,2_* of the RPA–CS emulsions exhibited no significant change (Figure 3a) and destabilization was not found after 7 days of storage, indicating that RPA–CS complexes had better emulsification properties than RPA. The PDI values of RPA–CS emulsions were less than that of RPA (Figure S2), declaring a narrow size distribution of RPA–CS emulsions after storage. This manifested that enough RPA–CS complexes were necessary to form a layer on the oil–water interface [30]. Similarly, the stability of emulsions coated with potato protein/CS complexes was also better than those coated with potato protein alone [8].

### 3.6. Microrheological Properties

**Figure 4:**
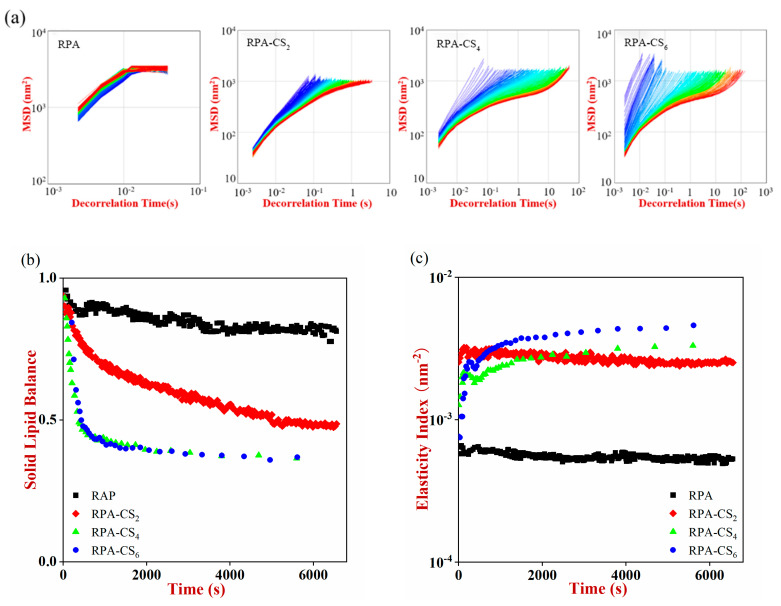

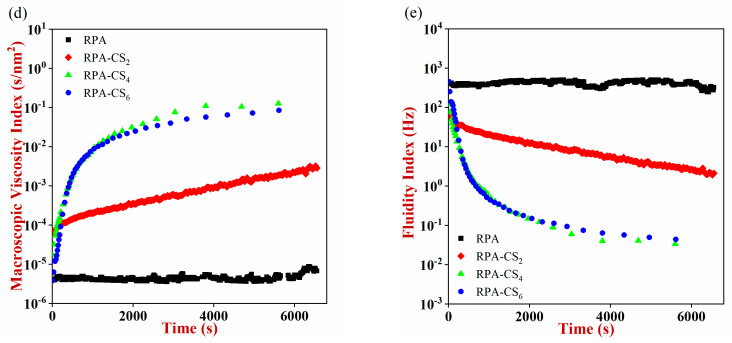
(**a**) The MSD profiles, Different colors changed from blue to red refer to different scanning time of MSD analysis. (**b**) solid lipid balance (SLB), (**c**) elasticity index (EI), (**d**) macroscopic viscosity index (MVI), and (**e**) fluidity index (FI) profiles of the emulsions.

Microrheology was used to measure the mean square displacement (MSD) curve of the emulsions, as it provides useful information about their local viscosity and elasticity. For viscoelastic fluids, the MSD curve should be nonlinear. Nonlinear MSD curves were observed for the RPA–CS complex emulsions (Figure 4a), indicating that they were non-Newtonian fluids with viscoelastic characteristics. The decorrelation time increased as the CS concentration increased, with the RPA–CS_6_ emulsions having the longest decorrelation time scale and lowest plateau value. This suggested that RPA–CS_6_ emulsions demonstrated the strongest viscoelastic properties [3].

The viscoelasticity of emulsions could also be measured by solid–liquid balance (SLB) values: SLB < 0.5 indicates predominantly elastic behavior; SLB = 0.5 indicates equal viscous and elastic behavior; and SLB > 0.5 indicates predominantly viscous behavior [30]. The SLB values gradually declined with time for all emulsions (Figure 4b), showing that the samples exhibited more solid-like behavior. The final SLB values of the RPA emulsions remained above 0.5, indicating their viscous behavior. The SLB values of all the RPA–CS emulsions were lower than 0.5, declaring their elastic behavior. The SLB values of the RPA–CS_4_ and RPA–CS_6_ emulsions decreased more rapidly than RPA–CS_2_ samples, indicating that higher CS concentrations led to more obvious elastic behavior.

The elasticity index (EI) is the inverse of the distance traveled by the droplet before it interacts with the network and indicates the elastic strength of the sample. The elasticity index of the emulsions decreased in the following order: RPA–CS_6_ > RPA–CS_4_ > RPA–CS_2_ > RPA (Figure 4c). The macroscopic viscosity index (MVI) is the inverse of the average velocity of the particles calculated over a long period and characterizes the macroscopic viscosity of the sample at zero shear. Typically, a higher MVI value leads to a more stable emulsion. The fluidity index (FI) is negatively correlated with the MVI value and provides a measure of the overall fluidity of the system. For the RPA emulsion, the values of EI, MVI, and FI remained constant (Figure 4c–e), indicating that the Brownian motion of the emulsion droplets was not restricted. For RPA–CS emulsions, EI and MVI increased with increasing CS concentration, whereas FI decreased. This phenomenon indicated that the RPA–CS emulsion droplets moved more slowly, which may be due to the interactions between the droplets [35].

### 3.7. In Vitro Simulation of Changes in Emulsions during Digestion

#### 3.7.1. Initial System

**Figure 5:**
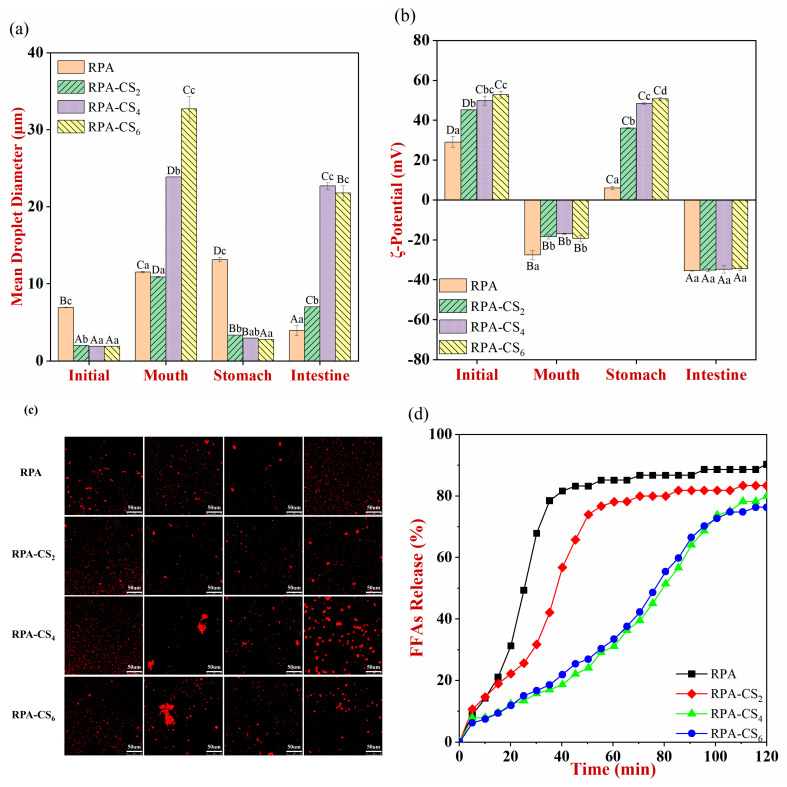

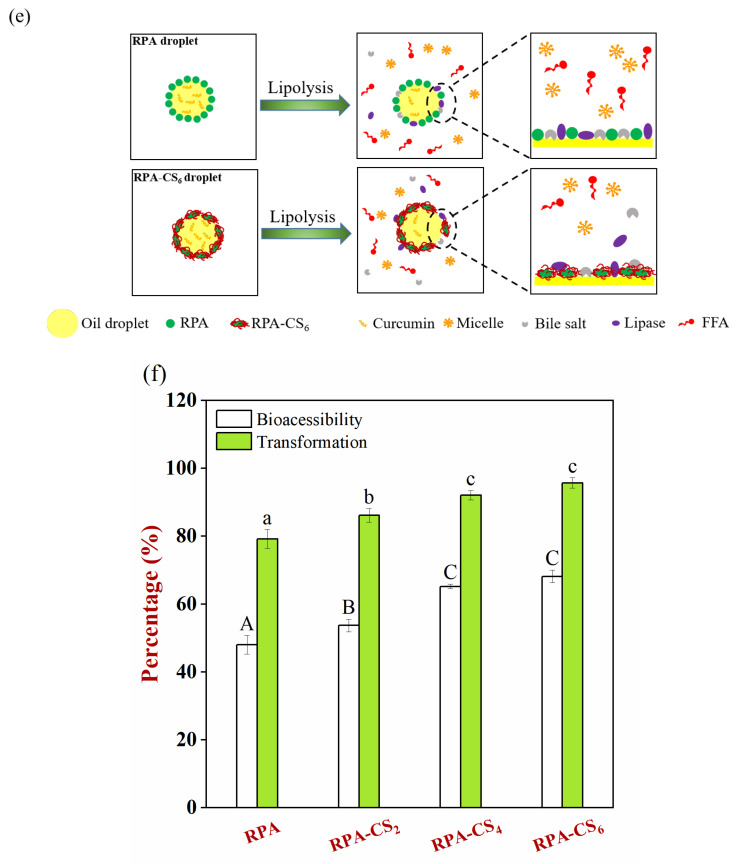
Physical properties of curcumin–loaded Pickering emulsions during simulated gastrointestinal tract digestion: (**a**) mean droplet diameter, (**b**) ζ–potential, (**c**) confocal microscopy images. Uppercase letters (A, B, C, D) and lowercase letters (a, b, c, d) indicate significant differences between different stages of digestion and different samples, respectively. (**d**) The free fatty acids released in Pickering emulsions. (**e**) Schematic illustration of lipolysis of curcumin–loaded emulsion. (**f**) The bioaccessibility and transformation of curcumin in Pickering emulsions.

The larger *d_3,2_* of the RPA emulsions (Figure 5a) might be due to its low water solubility under these conditions, leading to poor emulsifying properties. The incorporation of CS decreased the *d_3,2_* and increased the surface potential of oil droplets. A stronger electrostatic and steric repulsion for the interfacial layers formed from the RPA and CS might have prevented the aggregation of the oil droplets.

#### 3.7.2. Mouth Phase

The *d_3,2_* of all the emulsions increased significantly and the ζ–potential became negative after oral digestion (Figure 5b). This phenomenon was mainly caused by the changes in pH (from pH 3 in the initial emulsion to pH 6.8 in the oral phase). In addition, some negative charge might be because of the presence of anionic mucin in the SSFs. The mucin could adsorb to the oil droplet surfaces and accelerate their flocculation, thereby increasing the *d_3,2_* of the emulsions [36]. The CLSM images also declared that the emulsions contained relatively large droplets, especially for the RPA–CS_4_ and RPA–CS_6_ emulsions (Figure 5c). The droplet size of soy protein–polysaccharide-stabilized emulsions also increased after exposure to a simulated oral environment [35].

#### 3.7.3. Stomach Phase

After stomach digestion, the RPA–emulsion droplets showed an increased trend for *d_3,2_* with a low cationic charge of ζ–potential (Figure 5b), which indicated that appreciable droplet aggregation occurred [36]. There were several possible reasons for this effect. First, it might be because of the screening of the electrostatic repulsion between the oil droplets by mineral ions in the simulated gastric fluids. Second, it may be achieved by the bridging flocculation of the cationic oil droplets adsorbed by anionic mucin molecules. Third, it might be the result of pepsin hydrolysis of the adsorbed proteins, thereby declining the steric repulsion between the oil droplets [34].

The *d_3,2_* of all the RPA–CS emulsions were significantly smaller than the RPA emulsions, and the absolute values of their ζ-potential were higher. The CLSM images also showed that the presence of the RPA–CS complexes altered the aggregation state of the emulsion droplets in the gastric phase, with droplet aggregation being partially suppressed compared to RPA emulsions (Figure 5c). This behavior could be because the adsorbed complexes enhanced the electrostatic and steric repulsion between the oil droplets [36].

#### 3.7.4. Small Intestine Phase

After intestinal digestion, the decreased *d_3,2_* of RPA emulsions was mainly due to the fact that the oil droplets were gradually hydrolyzed by lipase [8]. In contrast, the increased droplet size with relatively large irregular-shaped particles could be found for RPA–CS emulsions (Figure 5c). This might be attributed to the fact that the pH of the small intestine was closer to the isoelectric point of RPA–CS complexes, thereby causing them to aggregate. The size of cellulose nanocrystal-stabilized Pickering emulsions also increased after the small intestinal stage [37]. After small intestinal digestion, all samples had a similar negative surface charge, which could be induced by the presence of colloidal particles comprised of anionic components like bile salts, peptides, and free fatty acids. Caseinate and glycoconjugate emulsions also had anionic ζ–potential values after exposure to the small intestinal phase [38].

### 3.8. Lipid Digestion Profiles

The RPA emulsion was initially digested rapidly, releasing approximately 70% of FFAs after 30 min, followed by a slow digestion, producing around 90% of FFAs after 120 min (Figure 5d). The extent of lipid digestion of the RPA–CS emulsions was dramatically lower than that of the RPA emulsions. The RPA–CS complexes adsorbed onto the oil droplet surfaces might hinder the contact between lipase and the droplet surfaces, thereby hindering lipid digestion (Figure 5e). The FFA release was also lower for hydrolyzed whey protein emulsions with the addition of polysaccharides than the one without polysaccharides. There was no significant difference in the FFA release between RPA–CS_4_ emulsions (79.8%) and RPA–CS_6_ emulsions (76.3%) [34]. Lipase needs to anchor to the oil–water interface before it can hydrolyze the lipids. The interfaces formed by RPA–CS_4_ and RPA–CS_6_ complexes might have been more effective at inhibiting lipase adsorption to the oil droplets, thereby reducing lipid digestion.

### 3.9. Curcumin Bioaccessibility and Transformation

The bioaccessibility and transformation of curcumin declined in the following subsequence: RPA–CS_6_ (68.2%) ≥ RPA–CS_4_ (65.2%) > RPA–CS_2_ (53.8%) > RPA (48.1%) (Figure 5f). These results indicated that the RPA–CS complexes prominently improved the curcumin bioaccessibility and stability. A similar behavior was pointed out by Hu et al. [35]. Presumably, the complexes formed a steric barrier that increased the stability of the curcumin. The RPA–CS_4_ and RPA–CS_6_ had a higher curcumin bioaccessibility than other samples, which might be due to their smaller particle size and the larger specific surface area of the droplets after gastric digestion, allowing more curcumin to be released into the mixed micelles, thus improving their bioaccessibility and stability [3].

## 4. Conclusions

RPA–CS complexes could be formed by coupled treatment of heat-assisted pH shifting and compounded CS. A uniform suspension was found for RPA–CS complexes at pH 2 and 3. The particle size of RPA decreased first and then increased with the addition of CS, with a smaller particle size of RPA–CS_2_. The complexes could be used to form oil-in-water emulsions that had improved stability compared to emulsions formed with RPA alone. The RPA–CS_4_ and RPA–CS_6_ emulsions enhanced the bioaccessibility and stability of curcumin under simulated gastrointestinal conditions compared with RPA emulsions. In the future, it will be important to show that these complexes perform well under commercial conditions and that they are economical to produce.

## Figures and Tables

**Table 1:** Slopes of D–f plots from the third overtone for samples during adsorption and rinsing at the hydrophobic gold surface.

Sample	K_1_ × 10^6^ (Hz^−1^)	K_2_ × 10^6^ (Hz^−1^)	K_3_ × 10^6^ (Hz^−1^)	K_4_ × 10^6^ (Hz^−1^)
RPA	0.112 ± 0.038 ^a^	0.122 ± 0.001 ^a^	0.299 ± 0.037 ^a^	0.084 ± 0.001 ^a^
RPA–CS_2_	0.331 ± 0.05 ^b^	0.104 ± 0.095 ^a^	0.667 ± 0.004 ^b^	0.143 ± 0.047 ^ab^
RPA–CS_4_	0.451 ± 0.004 ^c^	0.459 ± 0.084 ^b^	0.715 ± 0.061 ^b^	0.170 ± 0.013 ^b^
RPA–CS_6_	0.592 ± 0.018 ^d^	0.659 ± 0.002 ^c^	0.677 ± 0.001 ^b^	0.487 ± 0.026 ^c^

Values are given as the mean ± standard deviation. For each column, different letters (a–d) indicate significant differences (*p* < 0.05) between samples.

## Data Availability

Data is contained within the article or supplementary material.

## Supplementary Materials

The following supporting information can be downloaded at: https://www.mdpi.com/article/10.3390/foods12244384/s1.
