# Supplementary material for: Properties of Heat-Assisted pH Shifting and Compounded Chitosan from Insoluble Rice Peptide Precipitate and Its Application in the Curcumin-Loaded Pickering Emulsions
Source: Foods. 2023 Dec 6;12(24):4384. doi: 10.3390/foods12244384 (PMC10742475; doi:10.3390/foods12244384)
Supplement: Supplementary file 1 [file foods-12-04384-s001.zip › foods-2745302-supplementary.pdf]

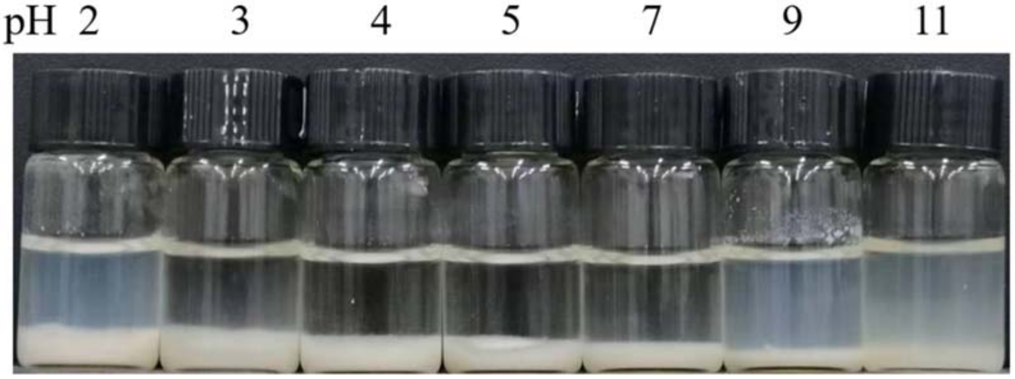

Figure S1 The appearance diagram of insoluble peptide precipitate at different pH.

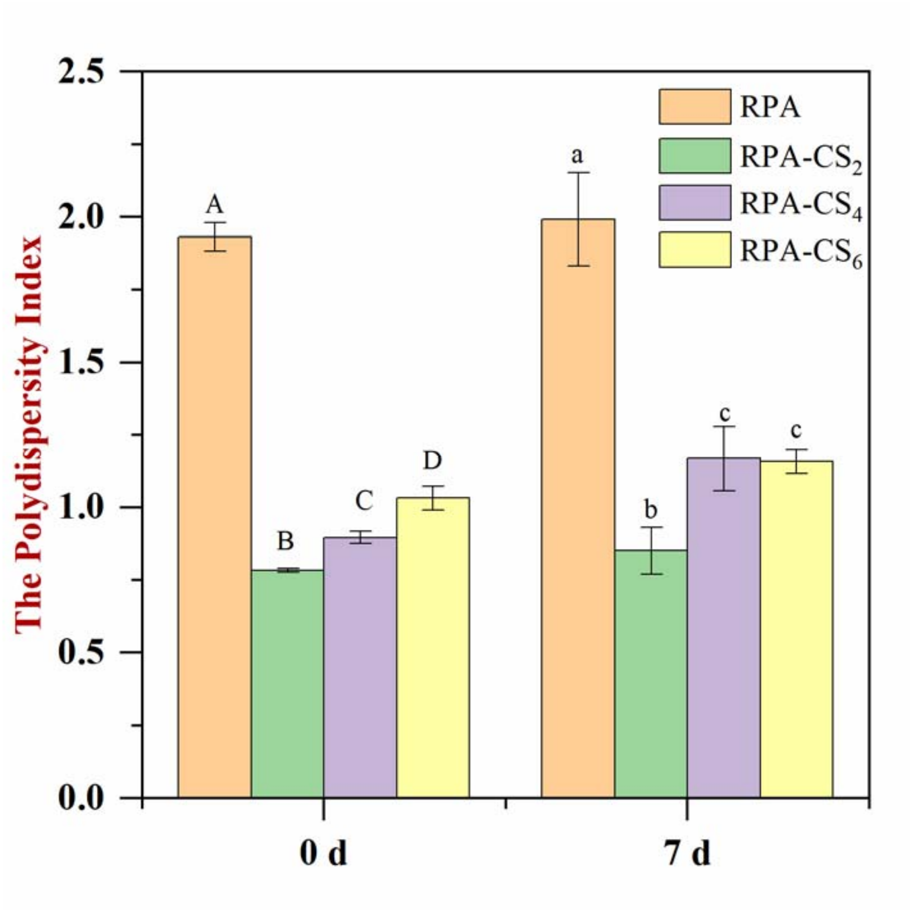

Figure S2 The polydispersity index of the emulsions. Different letters represent significant differences between groups (P < 0.05).
